# The effects of magainin 2-derived and rationally designed antimicrobial peptides on *Mycoplasma pneumoniae*

**DOI:** 10.1371/journal.pone.0261893

**Published:** 2022-01-24

**Authors:** Katsuhiko Hayashi, Takashi Misawa, Chihiro Goto, Yosuke Demizu, Yukiko Hara-Kudo, Yutaka Kikuchi

**Affiliations:** 1 Division of Microbiology, National Institute of Health Sciences, Kawasaki, Kanagawa, Japan; 2 Division of Organic Chemistry, National Institute of Health Sciences, Kawasaki, Kanagawa, Japan; 3 Graduate School of Medical Life Science, Yokohama City University, Yokohama, Kanagawa, Japan; Nanyang Technological University, SINGAPORE

## Abstract

Combating the spread of antimicrobial resistance (AMR) among bacteria requires a new class of antimicrobials, which desirably have a narrow spectrum because of their low propensity for the spread of AMR. Antimicrobial peptides (AMPs), which target the bacterial cell membrane, are promising seeds for novel antimicrobials because the cell membrane is essential for all cells. Previously, we reported the antimicrobial and haemolytic effects of a natural AMP, magainin 2 (Mag2), isolated from the skin of *Xenopus laevis* (the African clawed frog), four types of synthesised Mag2 derivatives, and three types of rationally designed AMPs on gram-positive and gram-negative bacteria. To identify novel antimicrobial seeds, we evaluated the effect of AMPs on *Mycoplasma pneumoniae*, which also exhibits AMR. We also evaluated the antimicrobial effects of an AMP, NK2A, which has been reported to have antimicrobial effects on *Mycoplasma bovis*, in addition to Mag2 and previously synthesised seven AMPs, on four strains of *M*. *pneumoniae* using colorimetric, biofilm, and killing assays. We found that three synthesised AMPs, namely 17base-Ac_6_c, 17base-Hybrid, and Block, had anti-*M*. *pneumoniae* (anti-Mp) effect at 8–30 μM, whereas others, including NK2A, did not have any such effect. For the further analysis, the membrane disruption activities of AMPs were measured by propidium iodide (PI) uptake assays, which suggested the direct interaction of AMPs to the cell membrane basically following the colorimetric, biofilm, and killing assay results. PI uptake assay, however, also showed the NK2A strong interaction to cell membrane, indicating unknown anti-Mp determinant factors related to the peptide sequences. Finally, we conclude that anti-Mp effect was not simply determined by the membrane disruption activities of AMPs, but also that the sequence of AMPs were important for killing of *M*. *pneumoniae*. These findings would be helpful for the development of AMPs for *M*. *pneumoniae*.

## Introduction

Antimicrobial resistance (AMR) is predicted to cause 10 million deaths worldwide in 2050 [1]. To combat this, the World Health Organization (WHO), the Food and Agriculture Organization of the United Nations (FAO), the World Organisation for Animal Health (OIE), government agencies, and pharmaceutical companies are promoting the One Health approach.

To prevent AMR, it is important to manage the usage of antimicrobials, and to develop and reserve drugs of last resort [2, 3]. Bacteria protect themselves from antimicrobials through gene mutations to produce an alternative antimicrobial-resistant target, degradation of antimicrobials, suppression of porin production, and production of multidrug efflux transporters [4]. It is essential to use adequate amounts of antimicrobials with appropriate methods, such as de-escalation treatment, which means changing drugs from broad to narrow spectrum by pathogens, but it is also necessary to develop new classes of antimicrobials that have a narrow spectrum and desirably a low potential to cause AMR [5].

Antimicrobial peptides (AMPs) are promising seeds for novel antimicrobials because they target the bacterial cell membrane, which is the most essential part of cells, including bacteria, and is seldom mutated for survival [6, 7]. For example, telavancin targets the membrane and the synthesis of peptidoglycan, and exhibits a slower rate of AMR development than rifampicin, which targets the ribosomes [8, 9]. The cell membrane is an ideal target for new antimicrobials, because any AMP-resistant cell membrane-related gene, which transfers horizontally, has not been reported, indicating a lower potential for AMR [10].

Natural AMPs, including magainin 2 (Mag2), which was isolated from the skin of *Xenopus laevis* (the African clawed frog), and their analogs or mimics are promising drug candidates [10]. We previously reported the characteristics and antimicrobial effects of Mag2 and of a series of synthesised Mag2-derived peptides, namely Mag2-17base, 17base-Aib, 17base-Ac_6_c, and 17base-Hybrid (Table 1) [11]. The Mag2 derivatives have amino acid insertions (2-aminoisobutyric acid (Aib), 1-aminocyclohexylcarboxylic acid (Ac_6_c), or lysine) in Mag2-17base, which is an essential part of Mag2 for the antimicrobial effect. We have also developed three rationally designed AMPs, namely Stripe, Random, and Block, with their helical structures and positions of positively charged lysine residues [12, 13]. The antimicrobial effect of these peptides on *Staphylococcus aureus*, *Staphylococcus epidermidis*, *Escherichia coli*, and *Pseudomonas aeruginosa* was evaluated [11–13].

**Table 1:** Sequences and properties of antimicrobial peptides.

Peptides	Amino acid sequences	Length	MIC (μmol/L)				Haemolysis (μmol/L)
			Gram-positive		Gram-negative		
			S. a.	S. e	E. c.	P. a.	
Mag2 [11]	H-GIGKFLHSAKKFGKAFVGEIMNS-NH_2_	23	100	25	3.13	12.5	>100
Mag2-17base [11]	H-GIGKFLHSAKKFGKAFV-NH_2_	17	50	12.5	3.13	3.13	>100
17base-Aib [11]	H-GIGKFLHSUKKFGKUFV-NH_2_	17	12.5	3.13	3.13	1.56	>100
17base-Ac_6_c [11]	H-GIGKFLHSZKKFGKZFV-NH_2_	17	3.13	3.13	3.13	6.25	3
17base-Hybrid [11]	H-GIKKFLKSZKKFVKZFK-NH_2_	17	3.13	1.56	3.13	3.13	3
Block [12, 13]	H-KKKKKKKKKGGGLLALLALLA-NH_2_	21	12.5	6.25	6.25	6.25	25
Stripe [12, 13]	H-KLLKKAGKLLKKAGKLLKKAG-NH_2_	21	12.5	1.56	3.13	1.56	>100
Random [12, 13]	H-KKKLAKLKLGAKLKLKGKLGA-NH_2_	21	100	6.25	12.5	3.13	100
NK2A [19]	H-TVIEVASKMCSKMRLLKGLCKSITKRFLRR-NH_2_	30	N.T.	N.T.	N.T.	N.T.	5

U, α-aminoisobutyric acid (Aib); Z, 1-aminocyclohexane-1-carboxylic acid (Ac_6_c); S. a., *Staphylococcus aureus*; S. e., *Staphylococcus epidermidis*; E. c.; *Escherichia coli*; P. a., *Pseudomonas aeruginosa*.

*Mycoplasma* spp. have an extraordinary cell membrane and lack the cell wall besides having one of the smallest known genomes; these are adaptations for a semi-parasitic life cycle [14]. *Mycoplasma pneumoniae* causes common cold-like symptoms with persistent dry cough, presenting as fulminant *M*. *pneumoniae* pneumonia in 1% of infected patients [15]. Owing to its unique cell membrane, Mycoplasma infections are treated with limited classes of antimicrobials—macrolides, quinolones/fluoroquinolones, and tetracyclines [16]. There has been an emergence of AMR in *M*. *pneumoniae*; for example, in Japan, 80%–90% of clinical isolates exhibit macrolide resistance [17].

In this study, we evaluated the anti-*M*. *pneumoniae* (anti-Mp) effects of Mag2, Mag2-17base, 17base-Aib, 17base-Ac_6_c, 17base-Hybrid, Stripe, Random, and Block for the antimicrobial developments. For verification of the methods used for evaluation, the Z′-factor, which is an evaluation score for screening systems, was used [18]. In addition to the Mag2 series, NK2A, an AMP that has been reported to be effective against *Mycoplasma bovis*, was also tested for its anti-Mp effect [19]. Finally, we performed propidium iodide (PI) uptake assay to evaluate the membrane disruption activity of AMPs [19].

## Materials and methods

### *M*. *pneumoniae* strains and culture conditions

*M*. *pneumoniae* FH (NBRC 14401) was purchased from the National Institute of Technology and Evaluation Biological Resource Center (Tokyo, Japan) [20–23]. *M*. *pneumoniae* strains M52, Mac, Bru, and M129-B7 (ATCC 15293, ATCC 15492, ATCC 15377, and ATCC 29342, respectively) were purchased from the American Type Culture Collection (Manassas, VA, USA). The culture medium was prepared by aseptically adding heat-inactivated horse serum (Biowest, Riverside, MO), fresh yeast extract (Nippon Beet Sugar Manufacturing, Tokyo, Japan), phenol red (Nacalai Tesque, Kyoto, Japan), and glucose (Nacalai Tesque) to final concentrations of 15%, 2%, 20 mg/L, and 3 g/L, respectively, to autoclaved Difco PPLO broth (BD Biosciences, Franklin Lakes, NJ, USA), and the pH was maintained between 7.6 and 7.8 with 1 mol/L sodium hydroxide. All subcultures were done in 75 or 150 cm^2^ flasks by incubating 0.2–0.3 mL/cm^2^ of inoculated broth culture at 37°C in a humidified atmosphere with 5% CO_2_. The culture was collected after 5–10 days of incubation in cryogenic tubes and stored below −80°C. For cell counting, two 5 μL aliquots of 10-fold serial dilutions were inoculated in triplicate on PPLO agar plates, which were prepared by aseptically adding heat-inactivated horse serum and fresh yeast extract to final concentration of 10% and 2% in autoclaved Difco PPLO agar (BD Biosciences). The plates were incubated for 7 or 10 days at 37°C to count and calculate the colony forming units (CFUs) by visualization under a stereoscopic microscope.

### Evaluation of anti-Mp effect of AMPs and sparfloxacin

The anti-Mp effect was evaluated using three methods: colorimetric and biofilm assays performed in 96-well plates, and killing assay performed on PPLO agar plates. Colorimetric and biofilm assays were performed using an initial density of 10^5^ CFU/mL, and the killing assay was performed using an initial concentration of 10^6^ CFU/mL, along with the un-inoculated blank. Sparfloxacin (SPFX; Sigma Aldrich) was used as a positive control and saline was used as a negative control. A mixture of 10 μL of saline-diluted antimicrobials or saline and 190 μL of culture medium per well was added to 96-well plates. After incubation for 6–8 days at 37°C with 0% or 5% CO_2_, colorimetric and biofilm assays were performed. In the colorimetric assay, the absorbance of the culture medium was measured at 560 nm using a Fusion α microplate reader (PerkinElmer, Waltham, MA, USA). In the biofilm assay, the biofilm at the bottom of the well was fixed with 10% formalin solution for 2 h [24], washed once with water, and then stained with 0.1% crystal violet solution for 10 to 15 min. The stained biofilm was washed twice with water, and was air-dried, and then the stain was extracted with 100 μL of 30% acetic acid. The absorbance of the content in the plate was measured at 550 nm using a Fusion α microplate reader. In the killing assay, 5 μL of antimicrobials at 20× final concentrations were prepared in sterilised saline and mixed with 95 μL of inoculated culture medium. After 3 days of incubation at 37°C, 10- and 100-fold culture dilutions were prepared in saline, and two 5 μL aliquots of each dilution were inoculated onto PPLO agar plates in triplicate. The CFU was counted after 7–10 days of incubation at 37°C under a stereoscopic microscope. The data were evaluated using the Welch’s *t* test or Shirley–Williams’ multiple comparison test and differences with a value of *p* < 0.005 were considered to be significant.

### Z′-factor calculation

The Z′-factor was used to evaluate the conditions of the colorimetric and biofilm assays. The Z′-factor for the results obtained for both the assays using media inoculated with 0, 10^3^, 10^4^, and 10^5^ CFU/mL of *M*. *pneumoniae* FH was calculated using formula (1), as previously described [18].


$$Z^{\prime }=1-\frac{\left[3\times Standard\;deviation\;(positive\;control)+3\times Standard\;deviation\;(negative\;control)\right]}{\left[Average\;(positive\;control)-Average\;(negative\;control)\right]}$$ (1)

### Peptide synthesis

Mag2 and its derivatives were synthesised using the Fmoc-based solid-phase method. The description of a representative coupling and deprotection cycle is presented here—The NovaPEG Rink amide resin was soaked for 30 min in CH_2_Cl_2_. After the resin was washed with dimethylformamide (DMF), Fmoc-amino acid (4 equivalents) and 2-(1*H*-benzotriazol-1-yl)-1,1,3,3-tetramethyluronium hexafluorophosphate (HBTU) (4 equivalents) dissolved in *N*-methyl-2-pyrrolidone (NMP) were added to the resin. *N*,*N*-Diisopropylethylamine (DIPEA) (4 equivalents) and 0.1 M hydroxybenzotriazole (HOBt) in NMP were added for the coupling reaction. The Fmoc-protective groups were deprotected using 20% piperidine in DMF. The resin was suspended in a cleavage cocktail (95% trifluoroacetic acid (TFA), 2.5% water, and 2.5% triisopropylsilane) at 20–25°C for 3 h. TFA was evaporated to a small volume under a stream of N_2_ and dripped in cold ether to precipitate the peptide. The peptides were dissolved in dimethylsulfoxide (DMSO) and purified using reverse-phase high-performance liquid chromatography using a Discovery^®^ BIO Wide Pore C18 column (25 cm × 21.2 mm; solvent A: 0.1% TFA/water, solvent B: 0.1% TFA/acetonitrile, flow rate: 10.0 mL/min, gradient: 10%–90% gradient of solvent B over 30 min). After purification, the peptide solution was lyophilised. The purity of peptides was assessed using analytical HPLC and a Discovery^®^ BIO Wide Pore C18 column (25 cm × 4.6 mm; solvent A: 0.1% TFA/water, solvent B: 0.1% TFA/acetonitrile, flow rate: 1.0 mL/min, gradient: 10%–100% gradient of solvent B over 30 min). Block, Stripe, and Random were synthesised as previously reported [12, 13]. NK2A was synthesised as previously reported [19]. All of the peptides were purified with volatile mobile phase. To assess again the purity and exact mass of the antimicrobial peptides, a Liquid Chromatograph-High Resolution Mass Spectrometry (LC-HRMS) analysis was used, operated with a shimadzu IT-TOF MS (Shimadzu, Kyoto, Japan) equipped with an electrospray ionization source. All peptide were dried by vacuum-lyophilization, and then prepared as aqueous solution by the dry weigh and calculated mass.

### Circular dichroism (CD) spectral analysis

CD spectrum was collected using a 1.0 mm path length cell in a J-1100 CD spectrometer (Jasco Corp., Tokyo, Japan) for each antimicrobial peptide solution: the PBS pH7.4-based solution containing 100 μmol/L peptide and 1% sodium dodecyl sulfate (SDS).

### Propidium iodide (PI) uptake assay

We performed the PI uptake assays based on the previously described method with modification for *M*. *pneumoniae* [19]. *M*. *pneumoniae* strains FH, M52, Mac, Bru, and M129-B7 were cultured as described above. Briefly, the 90 mL culture was done in PPLO-based medium for 6 days to observe the medium color change from red to yellow. The mycoplasma in the culture medium was centrifuged at 11,740*g* for 30 min at 4°C, and the precipitation was suspended in 20 mL phosphate buffered saline (PBS) by passing through a 60 mm-length 25-gauge needle (Top Corporation, Tokyo, Japan) three times. The suspension was centrifuged again at 11,740*g* for 30 min at 4°C, and 2.25 mL PBS was added to the precipitation. The 2× mycoplasma suspension was prepared by passing through a 60 mm-length 25-gauge needle five times. When using heat-killed *M*. *pneumoniae* (HKMp), the 2× suspension was heated at 95°C for 10 min. The 2× PI assay reaction mix based on PBS containing 20 μmol/L of an antimicrobial peptide or saline, and 10 μg/mL PI (Dojindo, Tokyo, Japan). Each assay was performed with a 96-well black flat-bottomed plate (PerkinElmer, Waltham, MA, USA) using a final cell volume of 100 μL prepared by mixing 2× mycoplasma suspension and 2× PI assay reaction mix at 1:1 in volume ratio. The intercalation fluorescence of PI was measured by using an Infinite 200 Pro F Plex microplate reader (Tecan, Zürich, Switzerland) every 2 min, with an excitation filter at 535 nm (half width: 25 nm) and an emission filter at 620 nm (half width: 20 nm). The averages and standard deviations of the fluorescent intensities was calculated, and normalised by an average fluorescence between 10 and 20 min of HKMp as 100% relative fluorescence, and by a minimum average fluorescence between 0 and 20 min of whole data as 0% relative fluorescence. The data were evaluated using the Welch’s *t* test with a value of *p* < 0.005 were considered to be significant.

## Results

### Synthesis and quality assessment of AMPs

We synthesised nine AMPs, and assessed the purity and mass by LC-HRMS, achieving the purities of 98.0%, 99.8%, >99.9%, 96.9%, 96.9%, 98.0%, >99.9%, >99.9%, and 96.5% for Mag2, MAG2-17base, 17base-Aib, 17base-Ac_6_c, 17base-Hybrid, Block, Stripe, Random, and NK2A, respectively (S1 Fig). The secondary structures were assessed by CD spectrum (S2 Fig), showing the typical α-helical patterns for all peptides [25].

### Evaluation of the effect of AMPs on *M*. *pneumoniae* FH strain using colorimetric and biofilm assays

**Fig 1:**
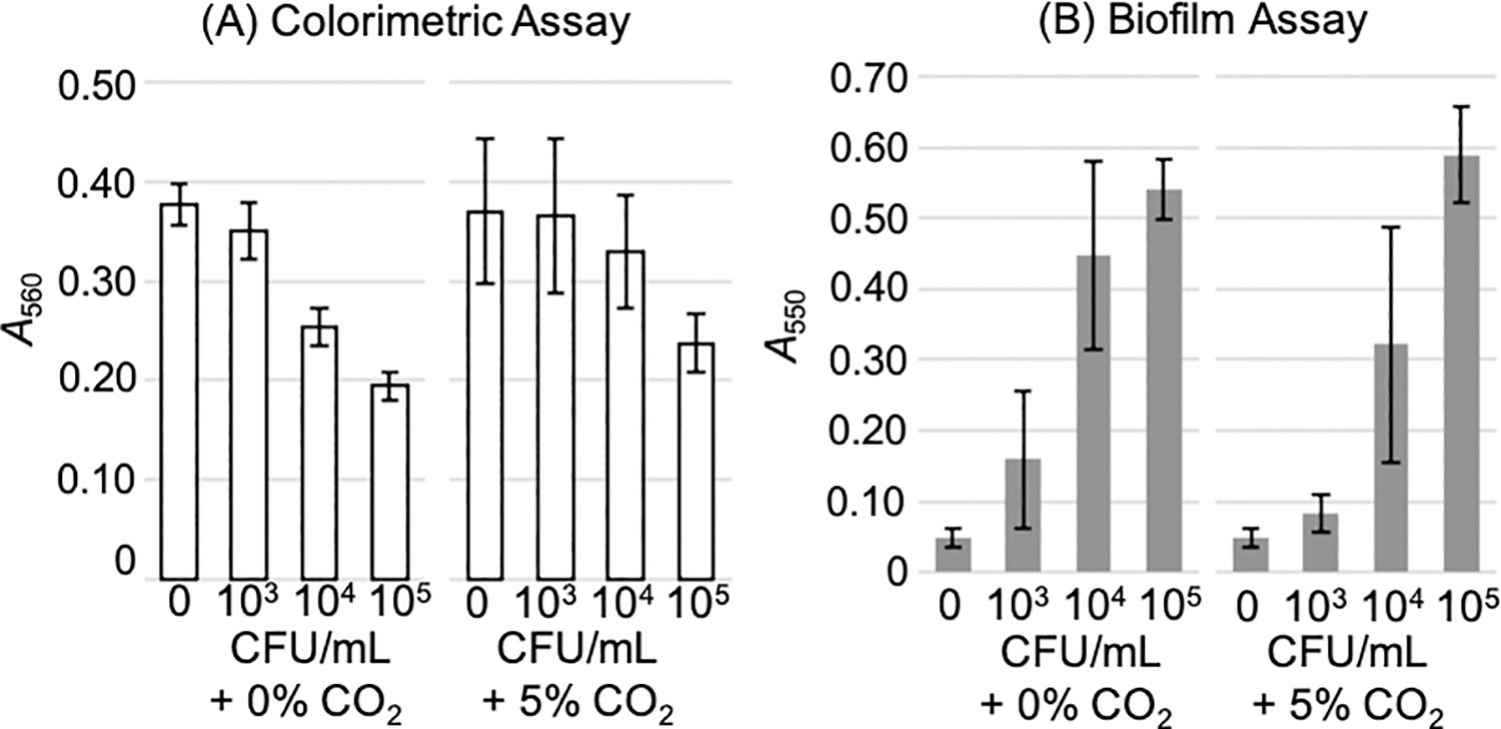
The results of colorimetric and biofilm assays under various initial conditions. *Mycoplasma pneumoniae* FH was inoculated at 10^3^, 10^4^, and 10^5^ CFU/mL in a 96-well plate and cultivated with 0% or 5% CO_2_. The growth was analysed by measuring the absorbance at 560 nm in the colorimetric assay (A) and at 550 nm in the biofilm assay (B). Error bars indicate ± 3 standard deviations.

First, to obtain evidence for the reliability of the methods, we evaluated the colorimetric assay and the biofilm assay under various conditions—the initial number of *M*. *pneumoniae* cells used (10^3^, 10^4^, or 10^5^ CFU/mL) and the culture conditions (with or without 5% CO_2_). The results are summarised in Fig 1. With 5% CO_2_, a sufficient reduction in the absorbance at 560 nm (colorimetric assay) was observed at 10^5^ CFU/mL, and sufficient production of biofilm was observed at 10^4^ CFU/mL. In the absence of CO_2_, a sufficient reduction in the absorbance was observed at 10^4^ CFU/mL, and sufficient production of biofilm was observed at 10^3^ CFU/mL. The errors with 5% CO_2_ were larger than those with 0% CO_2_. Because the stability and sensitivity with 0% CO_2_ were superior to those with 5% CO_2_, subsequent culture experiments were performed without CO_2_.

Next, we evaluated the effects of AMPs and SPFX (positive control) using the colorimetric assay and biofilm assay, at initial conditions of 10^5^ CFU/mL cells and 0% CO_2_. The complete repression of the growth was observed at concentrations of SPFX higher than 0.0625 μg/mL in the colorimetric assay (*p* = 0.0015 < 0.005), and than 0.25 μg/mL in the biofilm assay (*p* = 0.0048 < 0.005). Slight differences were found above 0.0625 μg/mL in the both assays.

**Fig 2:**
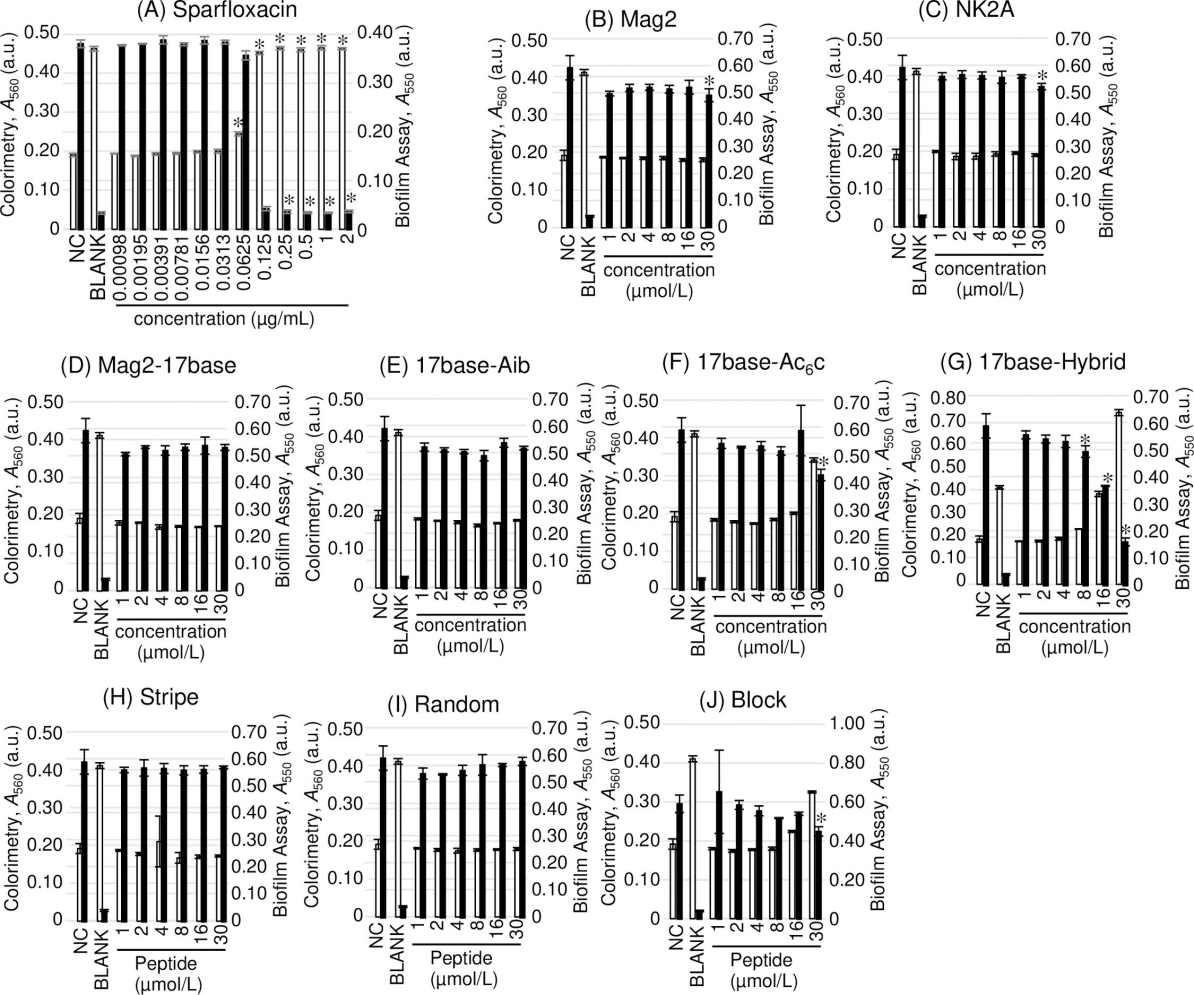
The anti-Mp effects of antimicrobials on *Mycoplasma pneumoniae* FH as evaluated by the colorimetric and biofilm assays. The colorimetric assay (white bar) and the biofilm assay (black bar) were used to evaluate the anti-Mp effects of sparfloxacin (A), and the antimicrobial peptides (AMPs) Mag2 (B), NK2A (C), MAG2-17base (D), 17base-Aib (E), 17base-Ac6c (F), 17base-Hybrid (G), Stripe (H), Random (I), and Block (J). NC means saline negative control and BLANK means the culture medium control without *M*. *pneumoniae* inoculation. Error bars indicate ± standard deviations. * indicates the statistical significance at *p* < 0.005 (Shirley–Williams’ multiple comparison test).

Using these methods, we evaluated the anti-Mp effects of the selected AMPs. Natural AMPs, Mag2 and NK2A, exhibited a statistically significant effect at 30 μmol/L (*p* = 0.0023, and 0.0023 < 0.005, respectively) in the biofilm assay, but no difference was observed in the colorimetric assay (Fig 2B and 2C). The four synthesised AMPs, Mag2-17base, 17base-Aib, Stripe, and Random (Fig 2D, 2E, 2H, and 2I), showed no difference in the biofilm assay (*p* = 0.014, 0.0086, 0.16, and 0.082 > 0.005, respectively). On the contrary, the other synthesised AMPs, 17base-Ac_6_c, 17base-Hybrid, and Block (Fig 2F, 2G and 2J), showed significant differences at concentrations of 30, 8, and 30 μmol/L in the biofilm assay (*p* = 0.0012, 0.0029 and 0.0017 < 0.005, respectively). At the high concentrations of 17base-Ac_6_c, 17base-Hybrid, and Block (Fig 2F, 2G, and 2J), the culture became muddy because of the formation of biofilm or degraded peptides, and the absorbance of the 17base-Hybrid at 30 μmol/L was higher than that of BLANK, which was the culture control without *M*. *pneumoniae*.

### The evaluation of the anti-Mp effects of AMPs using the killing assay

Next, we evaluated the number of surviving cells after treatment with antimicrobials. In the killing assay, we determined the CFU of surviving cells grown on agar plates. *M*. *pneumoniae* was preliminarily incubated for 1 h with antimicrobials as previously described for *M*. *bovis*, but the incubation with positive control SPFX, and nine AMPs showed no change in the CFU values (S3 Fig) [19]. We modified the method and used an incubation time from 1 h to 3 d. SPFX decreased the CFU value of *M*. *pneumoniae* in a dose-dependent manner (Table 2), and a significant difference was observed at concentrations above 0.0625 μg/mL (*p* = 0.0038 < 0.005). At 0.0625 μg/mL, SPFX treatment resulted in a decrease in the number of *M*. *pneumoniae* cells from the initially inoculated 10^6^ CFU/mL, which is equivalent to the results of the colorimetric and the biofilm assay. We applied this killing assay for evaluating the effects of AMPs.

**Table 2:** Validation of the killing assay with sparfloxacin.

Sparfloxacin (μg/mL)	0	0.0156	0.0313	0.0625	0.125	0.25
CFU/mL	1.47×10^7^	6.57×10^6^	2.33×10^6^	1.39×10^5^ ^*a*^	1.30×10^4^ ^*a*^	5.50×10^3^ ^*a*^
SD	2.04×10^5^	3.87×10^5^	2.00×10^4^	5.51×10^3^	1.00×10^3^	4.58×10^2^

^*a*^ *p* < 0.005 (Shirley–Williams’ multiple comparison test).

**Fig 3:**
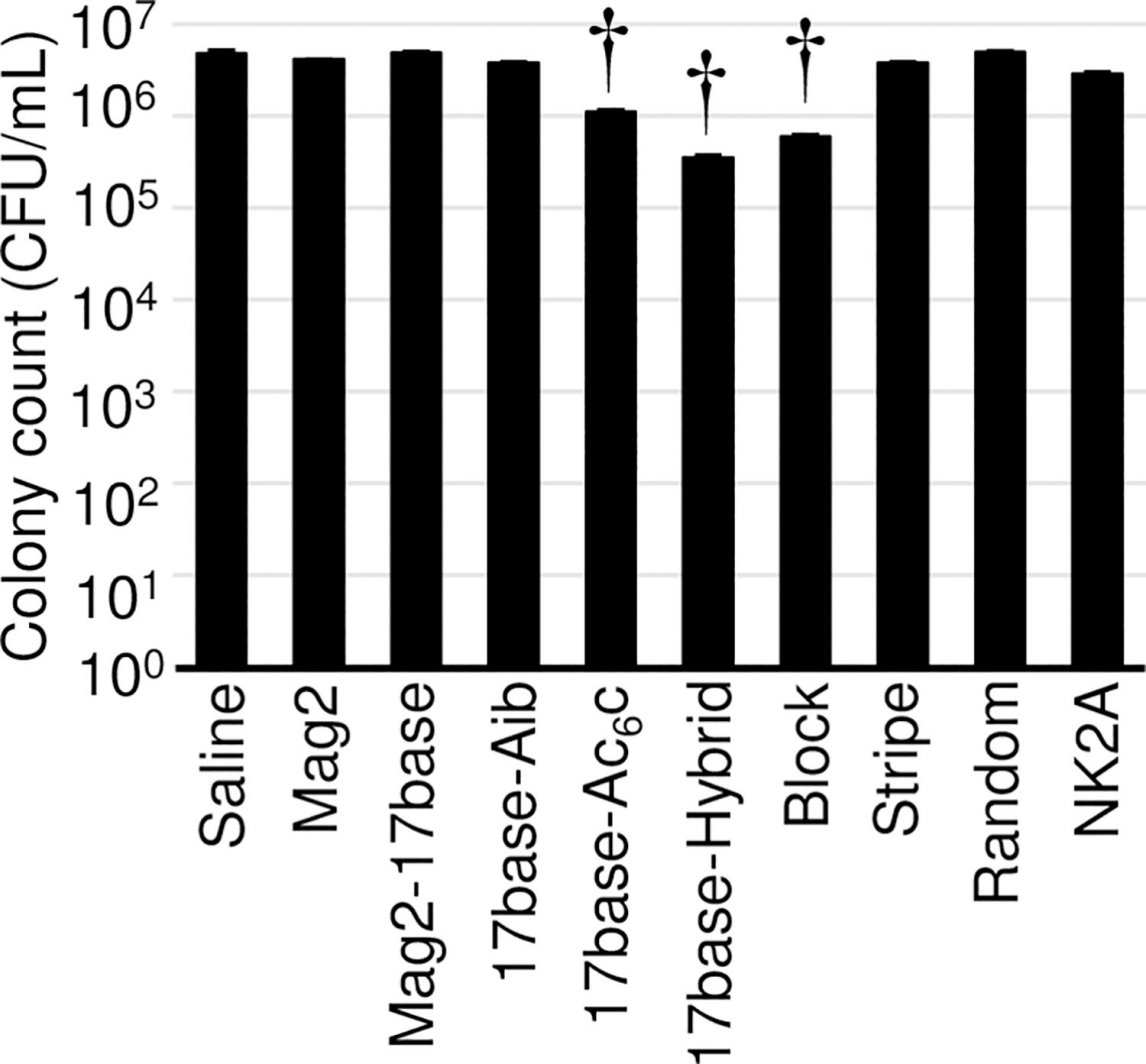
Evaluation of the antimicrobial effects of antimicrobial peptides (AMPs) on *Mycoplasma pneumoniae* FH using the killing assay. *M*. *pneumoniae* FH at 10^6^ CFU/mL were treated with 30 μmol/L AMPs (Mag2, Mag2-17base, 17base-Aib, 17base-Ac6c, 17base-Hybrid, Block, Stripe, Random, or NK2A) or saline for 3 days, and then inoculated onto PPLO agar in triplicate. The CFU values were determined as the number of surviving cells after 7 or 10 days of incubation. Error bars indicate ± standard deviations. † indicates the statistical significance vs. saline treatment at *p* < 0.005 (Welch’s *t* test).

The anti-Mp effects of AMPs determined using the killing assay are shown in Fig 3 and Table 3. Among the five bioactive AMPs, the number of cells treated with 17base-Ac_6_c, 17base-Hybrid, and Block showed a significant difference compared with that of cells treated with saline (*p* = 0.0023, 0.0016, and 0.0018 < 0.005, respectively); however, Mag2 and NK2A, as well as other AMPs, namely Mag2-17base, 17base-Aib, Stripe, and Random, had limited influence on the growth of *M*. *pneumoniae* (*p* = 0.056, 0.0093, 0.37, 0.016, 0.016, and 0.29 > 0.005, respectively). The reduction in the cell number was the largest upon treatment with 17base-Hybrid, followed in order by Block and 17base-Ac_6_c.

**Table 3:** The cell numbers after treatments with antimicrobial peptides in the killing assay.

Peptides or saline	Saline	Mag2	Mag2-17base	17base-Aib	17base-Ac_6_c ^a^	17base-Hybrid ^a^	Block ^a^	Stripe	Random	NK2A
CFU/mL	3.59×10^6^	4.06×10^6^	4.86×10^6^	3.75×10^6^	1.11×10^6^	3.50×10^5^	5.91×10^5^	3.71×10^6^	4.93×10^6^	2.87×10^6^
SD	4.36×10^5^	6.35×10^4^	1.82×10^5^	1.63×10^5^	4.51×10^4^	2.27×10^4^	2.67×10^4^	1.86×10^5^	1.56×10^5^	1.27×10^5^

^a^ *p* < 0.005 vs. saline treatment (Welch’s *t* test).

### Verification of the anti-Mp effects of AMPs using the four strains of *M*. *pneumoniae*

**Fig 4:**
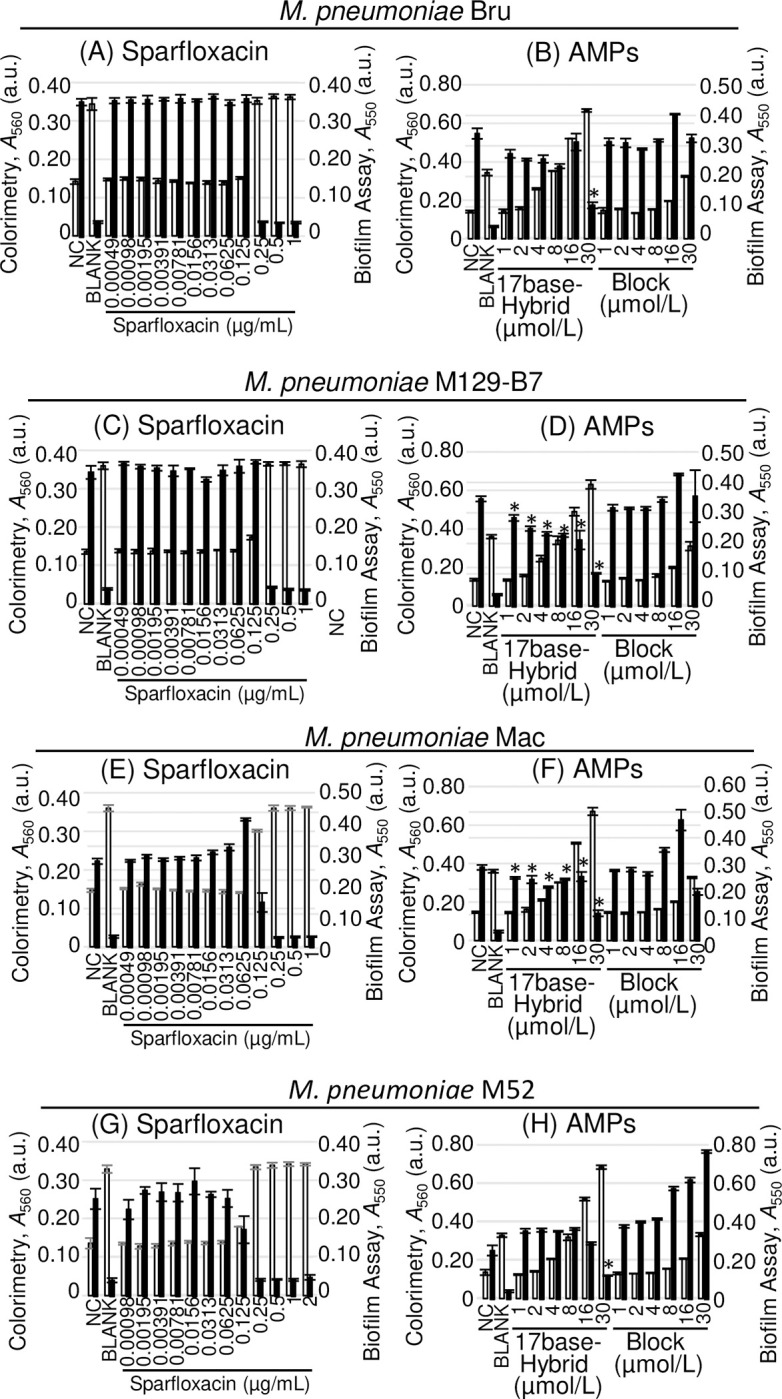
Evaluation of the antimicrobial effects of sparfloxacin and two antimicrobial peptides (AMPs; 17base-hybrid and block) on *Mycoplasma pneumoniae* strains Bru M129-B7, Mac, and M52. The results of the colorimetric assay (white bars) and biofilm assay (black bars) are depicted. *M*. *pneumoniae* Bru (A, B), M129-B7 (C, D), Mac (E, F) and M52 (G, H) were used for the tests, and sparfloxacin (A, C, E, G) was used as a positive control. The anti-Mp effects of 17base-Hybrid and Block (B, D, F, H) were verified. NC indicates saline negative control, and BLANK indicates the culture control without *M*. *pneumoniae*. Error bars indicate ± standard deviations. * indicates the statistical significance at *p* < 0.005 (Shirley–Williams’ multiple comparison test).

We evaluated the effects of two types of bioactive AMPs, 17base-Hybrid and Block, on four strains of *M*. *pneumonia*, namely Bru, M129-B7, Mac, and M52 [20–23]. Fig 4 shows the results of the colorimetric and biofilm assays with SPFX, 17base-Hybrid, and Block. The results of SPFX for the four strains of *M*. *pneumoniae* (Fig 4A, 4C, 4E and 4G) showed that 0.25 μg/mL of SPFX completely attenuated the growth, which indicates that the method can be applied to the *M*. *pneumoniae* strains universally. The results of the 17base-Hybrid peptide in the biofilm assay showed that this AMP had a statistically significant effect against Bru, M129-B7 and Mac strains at least at 30 μmol/L (*p* = 0.00044, 0.0023, and 0.0048 < 0.005, respectively), and a slight drop of biofilm production was observed on M52 strain (*p* = 0.096 > 0.005). Block peptide showed no statistically significant effect against four strains of *M*. *pneumoniae* (*p* = 0.10, 0.15, 0.0065, and 0.99 > 0.005 for strains Bru, M129-B7, Mac, and M52, respectively). Block peptide showed an enhancement of biofilm formation at 16 μmol/L in the four strains, and slight drop in the biofilm amount for the Bru and Mac strains, or no effect on the B129-B7 and M52 strains at 30 μmol/L (Fig 4B, 4D, 4F, and 4H).

**Fig 5:**
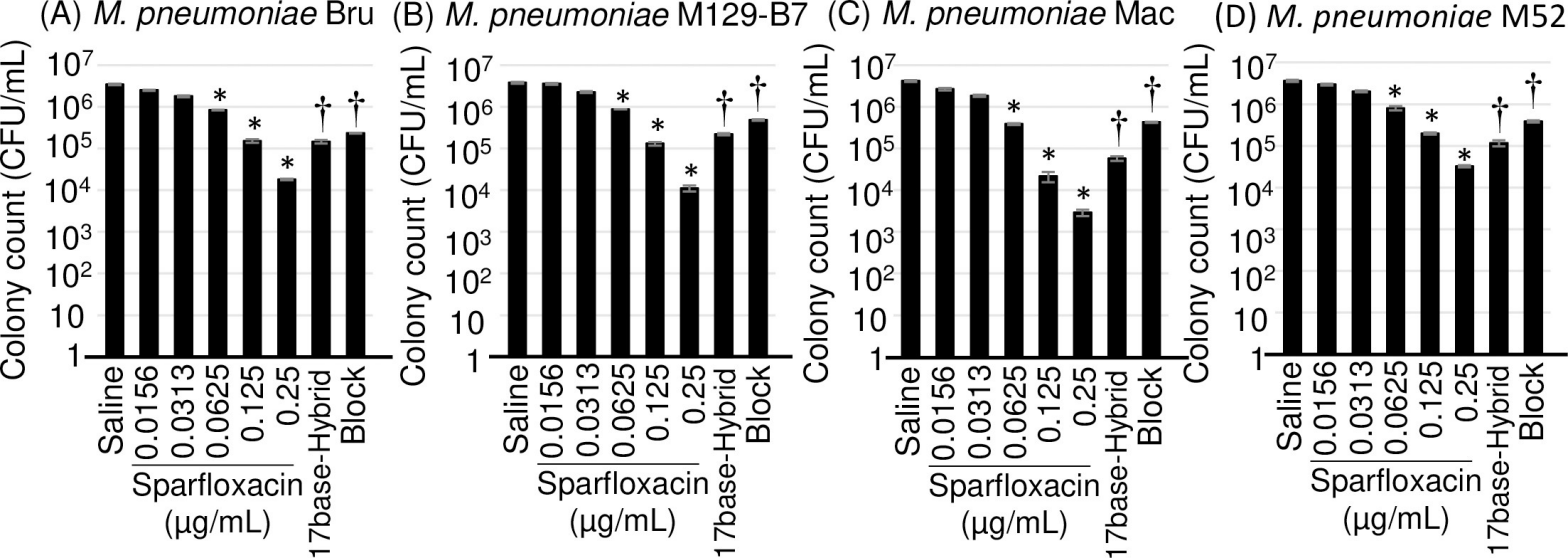
Evaluation of the effects of 17base-hybrid and block on *Mycoplasma pneumonia* strains Bru, M129-B7, Mac, and M52 using the killing assay. Four strains of *M*. *pneumoniae* at 10^6^ CFU/mL were treated with 30 μmol/L AMPs (17base-Hybrid and Block) or saline for 3 days, and inoculated onto PPLO agar in triplicate. The CFU values were determined as the number of surviving cells after 7 or 10 days incubation. Error bars indicate ± standard deviations. * indicates the statistical significance at *p* < 0.005 (Shirley–Williams’ multiple comparison test) and † indicates the statistical significance vs. saline treatment at *p* < 0.005 (Welch’s *t* test).

The results of the killing assay with SPFX, 17base-Hybrid, and Block are shown in Fig 5 and Table 4. The results of SPFX were statistically significant at 0.0625 μg/mL (*p* = 0.0038, 0.0047, 0.0037, and 0.0038 < 0.005 for strains Bru, M129-B7, Mac, and M52, respectively), and were lower than those of the colorimetric assay or the biofilm assay (0.25 μg/mL). 17base-Hybrid and Block showed a statistically significant decrease in the cell number when compared with the saline treatment (when using 17base-Hybrid, *p* = 0.00035, 0.00044, 0.00040, and 0.00064 < 0.005, and when using Block, *p* = 0.00036, 0.00051, 0.00048, and 0.00076 < 0.005, for strains Bru, M129-B7, Mac, and M52, respectively). The decrease in the cell number with 17base-Hybrid was larger than that with Block.

**Table 4:** Evaluation of the effects of sparfloxacin, 17base-hybrid, and block on four strains of *Mycoplasma pneumoniae* using the killing assay.

Strains		Sparfloxacin (μg/mL)						17base-Hybrid	Block
		0 (saline)	0.0156	0.0313	0.0625	0.125	0.25	30 μmol/L	30 μmol/L
Bru	Titre	3.48×10^6^	2.52×10^6^	1.80×10^6^	8.41×10^5^ ^a^	1.52×10^5^ ^a^	1.81×10^4^ ^a^	1.46×10^5^ ^b^	2.32×10^5^ ^b^
	(CFU/mL)								
	SD	1.51×10^5^	6.56×10^4^	9.50×10^4^	2.04×10^4^	1.55×10^4^	6.56×10^2^	1.50×10^4^	3.61×10^3^
M129-B7	Titre	3.79×10^6^	3.57×10^6^	2.26×10^6^	8.73×10^5^ ^a^	1.31×10^5^ ^a^	1.12×10^4^ ^a^	2.21×10^5^ ^b^	4.84×10^5^ ^b^
	(CFU/mL)								
	SD	1.83×10^5^	1.46×10^5^	9.71×10^4^	5.77×10^3^	1.27×10^4^	1.86×10^3^	1.31×10^4^	1.95×10^4^
Mac	Titre	4.19×10^6^	2.66×10^6^	1.86×10^6^	3.88×10^5^ ^a^	2.17×10^4^ ^a^	2.90×10^3^ ^a^	5.80×10^4^ ^b^	4.30×10^5^ ^b^
	(CFU/mL)								
	SD	2.02×10^5^	2.01×10^5^	1.16×10^5^	1.76×10^4^	6.03×10^3^	5.29×10^2^	7.21×10^3^	1.31×10^4^
M52	Titre	3.62×10^6^	2.95×10^6^	2.02×10^6^	8.00×10^5^ ^a^	1.99×10^5^ ^a^	3.24×10^4^ ^a^	1.16×10^5^ ^b^	3.89×10^5^ ^b^
	(CFU/mL)								
	SD	2.17×10^5^	1.31×10^5^	1.01×10^5^	9.17×10^4^	1.20×10^4^	1.99×10^3^	1.90×10^4^	2.22×10^4^

^a^ *p* < 0.005 (Shirley–Williams’ multiple comparison test)

^b^ *p* < 0.005 vs. saline treatment (Welch’s *t* test).

### The effects of AMPs to mycoplasma cell membrane using five strains of *M*. *pneumoniae*

**Fig 6:**
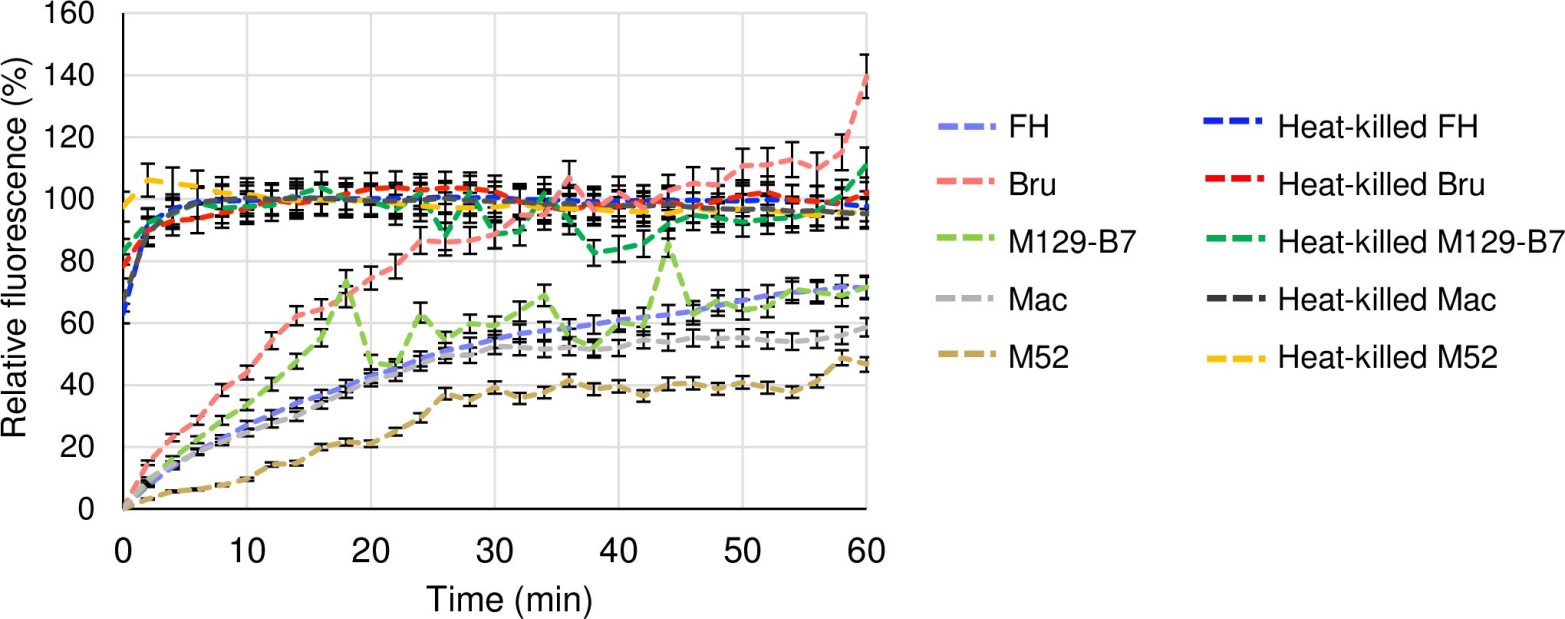
Results of propidium iodide (PI) uptake assays using heat-killed *Mycoplasma pneumoniae* (HKMp) or live *M*. *pneumoniae*. A suspension of live or heat-killed (95°C for 10 minutes) *M*. *pneumoniae* strains FH (pale blue or blue dotted-lines), Bru (pale-red or red dotted-lines), M129-B7 (pale-green or green dotted-lines), Mac (grey or black dotted-lines), or M52 (yellow-ocher or yellow dotted-lines) in phosphate buffered saline pH 7.4 was mixed with PI at the final concentration of 5 μg/mL in triplicate, and the intercalate fluorescence (excitation at 535 nm, and emission at 620 nm) were immediately measured in every 2 min. The data was normalised using the averaged fluorescence as 100% between 10 and 20 min by each strain of HKMp. Error bars indicate ± standard deviations.

We evaluated the effects of AMPs to mycoplasma cell membrane by PI uptake assays. PI penetrating the membrane labels the cells by intercalating the genomic DNA, which distinguish the dead or membrane disrupted cells. The measurements of PI uptake using live *M*. *pneumoniae* (live Mp) or HKMp without any peptides showed the fluorescent differences (Fig 6). The fluorescence of each HKMp strain rose in 10 min, and was stable; on the other hand, the fluorescence of live Mp gradually rose over time. The difference between live Mp and HKMp was shrunk with time, and the significances of the fluorescence were observed until 18, 18, 16, 24, 58, and 24 min with their *p* values < 0.005 at 0.0047, 0.0015, 0.0026, and 0.0015 for *M*. *pneumoniae* strains FH, Bru, M129-B7, Mac, and M52, respectively. After 16 min from the start of measurement, the fluorescence was fluctuated especially in *M*. *pneumoniae* strain M129-B7. For the evaluation of AMP effects to the membrane, we used the fluorescence of PI uptake assays up to 20 min from the start of measurements.

**Fig 7:**
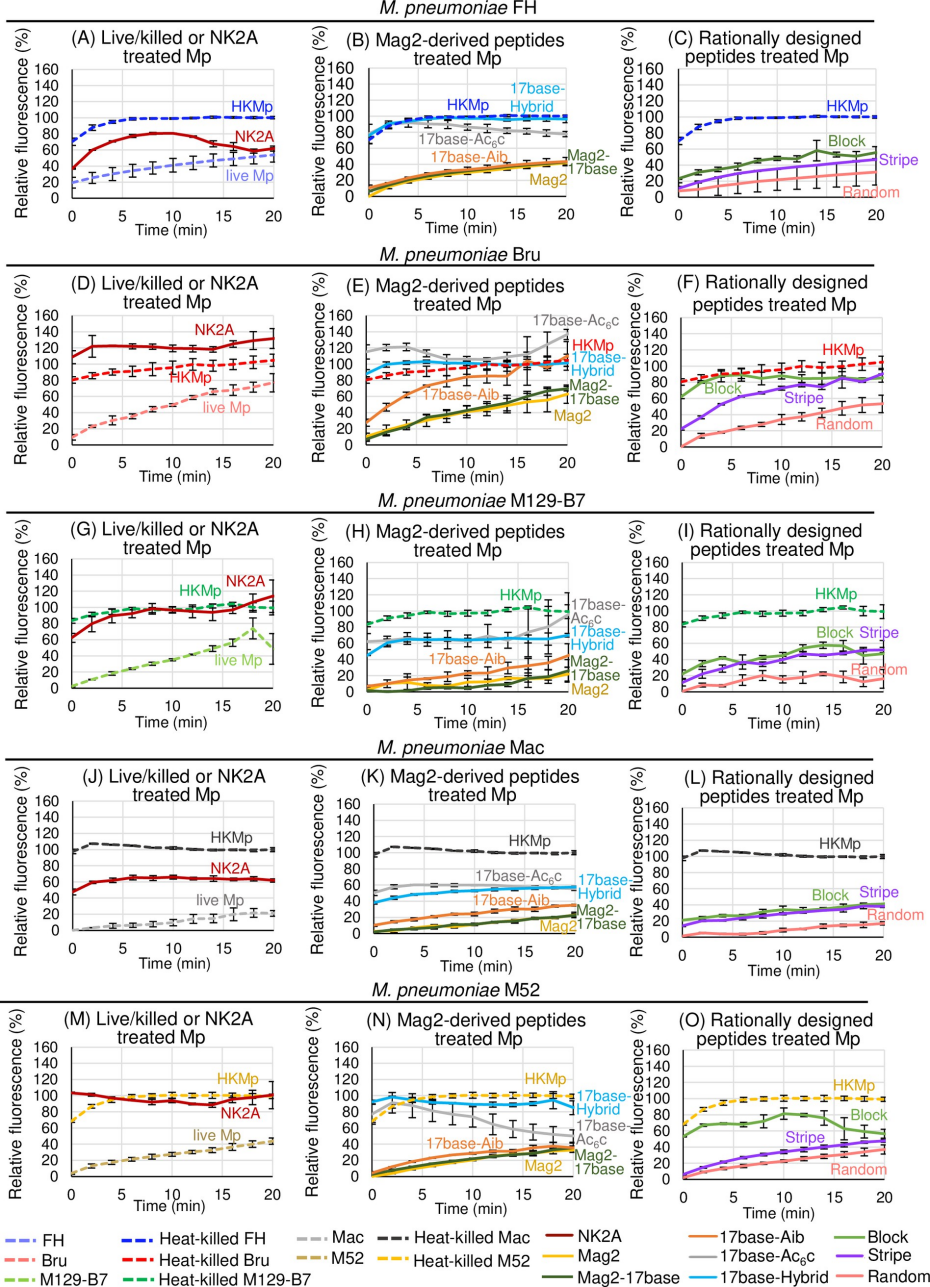
Results of propidium iodide (PI) uptake assays for the evaluation of the antimicrobial peptides effects to the mycoplasma cell membrane. A suspension of live *Mycoplasma pneumoniae* strains FH (A, B, and C), Bru (D, E, and F), M129-B7 (G, H, and I), Mac (J, K, and L), and M52 (M, N, and O) in phosphate buffered saline was mixed with PI (final 5 μg/mL) and antimicrobial peptides (final 10 μmol/L) in triplicate using NK2A (red-coloured line in panels A, D, G, J, and M), Mag2, Mag2-17base, 17base-Aib, 17base-Ac_6_c, 17base-Hybrid (yellow, dark-green, orange, grey, and light-blue lines respectively in panels B, E, H, K, and N), Block, Stripe, and Random (green, purple, and pink lines respectively in panels C, F, I, L, and O). The intercalate fluorescence of PI (excitation at 535 nm, and emission at 620 nm) were immediately measured in every 2 min. For the reference, the live or heat-killed *M*. *pneumoniae* (live Mp or HKMp) were also used without any antimicrobial peptides; live Mp of strains FH, Bru, M129-B7, Mac, and M52 were depicted by pale-blue, pale-red, pale-green, grey, yellow-ocher dotted-lines respectively in panels A, D, G, J, and M, and HKMp of strains FH, Bru, M129-B7, Mac, and M52 were depicted by blue, red, green, black, yellow dotted-lines respectively in all panels. The data was normalised using the averaged fluorescence from 10 to 20 min by each strain of HKMp. Error bars indicate ± standard deviations.

We used five strains of *M*. *pneumoniae* with 10 μmol/L AMPs for PI uptake assays, and the results were summarised in Fig 7. When using NK2A, the PI fluorescence arose in five strains (Fig 7A, 7D, 7G, 7J, and 7M) when comparing to the results of live Mp. When using Mag2-derived peptides, the fluorescence arose for 17base-Hybrid and 17base-Ac_6_c, and slight fluorescent shift was observed 17base-Aib especially in *M*. *pneumoniae* Bru (Fig 7B, 7E, 7H, 7K, and 7N). When using Mag2-derived peptides, the fluorescence arose in the order of Block, Stripe, and Random from the 1st to 3rd place (Fig 7C, 7F, 7I, 7L, and 7O). The fluorescence of live Mp without any peptides were higher than that with Mag2, Mag2-17base, and Random in five strains, and than that with 17base-Aib, and Stripe in strains FH, M129-B7, and M52 (Fig 7B, 7C, 7E, 7F, 7H, 7I, 7K, 7L, 7N, and 7O).

## Discussion

In this study, we assessed the anti-Mp effects of AMPs. Conventionally, minimum inhibitory concentration (MIC) values are determined using a colorimetric assay in 96-well plates by measuring the change in colour of the culture medium from red to yellow, which is caused by the production of acetic acid along with the growth of *M*. *pneumoniae*, by supplemented pH indicator, for example phenol red [26]. Because the maximum fluctuation range for the absorbance of phenol red is at 560 nm in association with the pH shift, the reduction in the absorbance at 560 nm indicates the growth of *M*. *pneumoniae*. We used a biofilm assay to evaluate the anti-Mp effect in addition to the colorimetric assay. Biofilm assays are usually used to quantify biofilms, which are one of the AMR factors [27, 28].

The sensitivities of the colorimetric and biofilm assays can be compared using the Z′-factor [18]. The Z′-factor is generally used for the evaluation of the screening system, indicating the size of the band gap between the positive and negative controls. A Z′-factor above 0.5 indicates a good detection system. The calculated Z′-factor for the initial CFUs of 10^5^ and 10^3^ were 0.73 and 0.64 for the colorimetric assay and biofilm assay without CO_2_ (Fig 1). The Z′-factors for the colorimetric and biofilm assays suggested that both these tests can detect the effect of antimicrobials.

Both the conventional colorimetric assay and biofilm assay gave clear results with the positive control, SPFX (Figs 2A, 4A, 4C, 4E, and 4G) at 0.0625–0.250 μg/mL, as previously reported [29, 30]. However, with AMPs, the muddy precipitate that was formed interfered with the absorbance at 560 nm in the colorimetric assay, resulting in the failure of the assay (Figs 2B, 2C, 2F, 2G, 2J, 4B, 4D, 4F, and 4G). The muddy precipitation seemed to occur when AMPs affected the growth of *M*. *pneumoniae*. The biofilm assay washed out the precipitate to evaluate the antimicrobial effects of AMPs on *M*. *pneumoniae* adequately. Because of the interference in the results of the colorimetric assay, we adopted the results of the biofilm assay as the anti-Mp effects of AMPs.

The anti-Mp effects of Mag2, NK2A, 17base-Ac_6_c, 17base-Hybrid, and Block were detected using the biofilm assay with *M*. *pneumoniae* FH (Fig 2B, 2C, 2F, 2G, and 2J); however, Mag2-17base, 17base-Aib, Stripe, and Random did not show any anti-Mp effects (Fig 2D, 2E, 2H, and 2I). The anti-Mp effects of 17base-Ac_6_c, 17base-Hybrid, and Block were proven with the killing assay using *M*. *pneumoniae* FH (Table 3; Fig 3). On the contrary, Mag2 and NK2A did not show their killing effect even at 30 μmol/L (Table 3; Fig 3). 17base-Ac_6_c, 17base-Hybrid, and Block appeared to possess anti-Mp effects, but those of Mag2 and NK2A appeared to be limited.

Further analysis using the four strains of *M*. *pneumoniae* showed that 17base-Hybrid and Block possess anti-Mp activities universally (Table 4; Figs 4B, 4D, 4F, 4H, and 5). Block peptide has no statistically significant effect, but the enhancement of biofilm production was observed in the four strains of *M*. *pneumoniae* (Fig 4), which was also observed with *M*. *pneumoniae* FH (Fig 2F and 2J), indicating the anti-Mp effect at higher concentrations (>30 μmol/L). The killing assay showed that both 17base-Hybrid and Block AMPs had anti-Mp effects (Table 4; Fig 5). Because in the killing assay, the four strains of *M*. *pneumoniae* cannot be killed completely at MICs even when using SPFX, whether the anti-Mp effect is bacteriostatic or bactericidal cannot be distinguished. However, it is clear that 17base-Hybrid and Block, and possibly 17base-Ac6c, possess at least bacteriostatic effects (Fig 5).

All the AMPs used in this study have been reported to have antimicrobial effects [11–13]. Mag2 is least effective against *S*. *epidermidis*, *E*. *coli*, and *P*. *aeruginosa* at concentrations from 3.13 to 25 μmol/L, and the synthesised Mag2 series (Mag2-17base, 17base-Aib, 17base-Ac_6_c, and 17base-Hybrid) showed lower MICs for these bacteria compared with those of Mag2 (Table 1) [11]. Stripe, Random, and Block showed antimicrobial effects at 1.56 to 12.5 μmol/L concentrations [12, 13]. NK2A was also reported as an effective AMP against bovine pathogen bacteria, *Histophilus somni*, *Pasteurella multocida*, and *Mannheimia haemolytica* at 5 μmol/L, and against *M*. *bovis* at 20 μmol/L [19].

The haemolytic effect, which cannot be separated from the antimicrobial effect of AMP, partly accounts for the anti-Mp effects. Among the AMPs used, 17base-Ac_6_c, 17base-Hybrid, and Block, which were active, and NK2A, which was inactive, had haemolytic effects at 3, 3, 25, and 5 μmol/L (Table 1) [11–13]. In the Mag2 series, the bulky Ac_6_c residue stabilises the alpha-helix structure to penetrate the cell membrane, which enhances the haemolytic effect [13]. The CD spectrum showed the stabilisation of the alpha-helical structure in the order of 17base-Hybrid, 17base-Ac_6_c, 17base-Aib, and Mag2-17base from the 1st to 4th place based on the signals between 190 nm and 200 nm in Mag2-derived AMPs (S2A Fig.). These data supported the anti-Mp effects of Mag2-derived AMPs (Figs 2D, 2E, 2F, 3G, and 3). On the other hand, in the rationally designed AMPs, Block peptide showed the strongest anti-Mp activity in spite of the weakest stabilisation of alpha-helical structure, indicating that a sequence dependency for anti-Mp effect exists in addition to alpha-helical stabilisation (Table 1; Figs 2J and S2B). For NK2A, the anti-*M*. *bovis* effect was observed at 20 μmol/L, and there was a 4-fold difference in the effective concentration for haemolysis and for the anti-*M*. *bovis* effect, indicating selective antimicrobial activity against pathogens [19]. The CD spectra of NK2A showed the strongest stabilisation of the alpha-helical structure among the tested AMPs in spite of almost no anti-Mp effect (Figs 2C and S2B). The AMP structure stabilisation partly accounts for anti-Mp activity which was indicated by the results of Mag2-derived peptides, but there is also the AMP sequence dependency which was suggested by the results of the rationally designed AMPs and NK2A. To reveal the AMP structure-activity relationship for anti-Mp effect, we evaluated the AMPs membrane disruption activities by the PI uptake assay [19]. PI is the cationic intercalator which inserts the double-stranded DNA when entering cells. Ethidium bromide, which molecular framework resembles to PI, was suggested to be expelled by active efflux pumps, and PI would be also expelled from the mycoplasma cells [31]. Results of PI uptake assays using live Mp tend to fluctuate after 20 min from the start of measurements in spite of the stable results of HKMp, and the statistical significances (*p* < 0.005) between live Mp and HKMp were observed at least until 16 min (Fig 6). These results indicated the active efflux or other factor to resist xenobiotics including PI. The PI uptake assay results shown by Dassanayake *et al*. which indicated no increase of PI fluorescence when using live *M*. *bovis* at final 3 μmol/L which equals to 2 μg/mL, but our PI uptake assay data with final 5 μg/mL showed the higher baselines [19]. Because the slight condition difference affected the baselines of intercalation fluorescence when ethidium bromide assays of *E*. *coli*, the difference of the PI uptake assay conditions would affect the baselines of our results [32, 33]. We used the data up to 20 min for the evaluation of membrane disruption activities to omit any effects of resistant reaction.

PI uptake assay results of *M*. *pneumoniae* FH showed that 17base-Hybrid, 17base-Ac_6_c, and Block, which showed anti-Mp effects, exhibited the relatively higher fluorescence in Mag2-derived peptide or rationally designed peptide groups (Figs 2F, 2G, 2J, 7B, and 7C). Because the higher fluorescence indicated the pore forming on the cell membrane, 17base-Hybrid, 17base-Ac_6_c, and Block have better activity for membrane disruption. When using Mag2, Mag2-17base, 17base-Aib, Stripe, or Random for *M*. *pneumoniae* FH, the fluorescence was suppressed than that of live Mp (Fig 7B, and 7C). Because AMPs possesses the cationic moiety to interact to cell membrane, the fluorescent suppression suggested the AMP-membrane interaction changing the electrical potential from negative to positive, resulting in dismissing of the cationic PI molecules. Other strains of *M*. *pneumoniae* also exhibited these fluorescent suppressions (Fig 7E, 7F, 7H, 7I, 7K, 7L, 7N, and 7O). Because 17base-Ac_6_c, 17base-Hybrid, and Block increased the fluorescent than live Mp strain FH with negative control, the increase of fluorescence than that of live Mp could be an indicator for the anti-Mp effect.

We compared the AMP membrane disruption activity in the Mag2-derived peptide and rationally designed peptide groups, because the charge and residue positions were well designed suitable for comparing. In Mag2-derived peptides, the membrane disruption activity of 17base-Hybrid was strongest and 17base-Ac_6_c followed next, which tendency was also observed in other *M*. *pneumoniae* strains, and slight effects of 17base-Aib were additionally observed (Fig 7E, 7H, 7K, and 7N). When comparing 17base-Aib, 17base-Ac_6_c, and 17base-Hybrid, the membrane disruption activity seemed to be affected by the structure stability, but strikingly, the N-terminal lysin residue seemed to be a key to empower the membrane disruption activity (Table 1; S2A Fig). In rationally designed peptides, there was a tendency that Block caused the highest fluorescence, indicating that Block has the strongest membrane disruption activity to mycoplasma membrane (Fig 7C, 7F, 7I, 7L, and 7O). The power of the membrane disruption activity seemed not to be support by the alpha-helical stability as described in the relationship with the anti-Mp effect (Figs 2H, 2I, 2J, and 3 and S2B). Because Block also has the lysin residues around N-terminus as well as 17base-Hybrid, the terminal cationic charge is a key for anti-Mp effect and membrane disruption activity. Our previous report showed that Block peptide had the weakest membrane disruption activity on bacteria, using the liposome mimicking gram-positive or gram-negative bacteria, and that the stabilisation of alpha-helical structure of AMP is a key for the antimicrobial activity to gram-positive or gram-negative bacteria [11, 12]. The lipid component of mycoplasma is different from that of other bacteria. Mycoplasma lacks the cell wall components i.e., peptidoglycan, Lipid II, lipopolysaccharides etc.; lacking these components are better factors for AMPs because steric hindrance can be avoided when approaching [6]. Mycoplasma also have a rich concentration of cardiolipin, which could protect the membrane by stabilization of curvature [34]. Because the cationic property is important for the approach to the membrane and the structural stability is important for the membrane disruption by the insertion, the predominance of the cationic moiety at the terminus over the alpha-helical stability could be partly explained by the membrane difference between mycoplasma and most other bacteria.

In spite of almost no anti-Mp effect, NK2A peptide showed the strong membrane disruption activities to five strains of *M*. *pneumoniae* in PI uptake assays (Figs 2B, 3, 7A, 7D, 7G, 7J, and 7M). NK2A have the cationic arginine residues at the C-terminus, which is a factor to contribute the membrane disruption activity (Table 1). If the anti-Mp effect was caused only by the membrane disruption activity, AMPs would kill *M*. *pneumoniae* in a short time, but neither NK2A, Block nor 17base-Hybrid can kill *M*. *pneumoniae* FH with 1 h AMP treatments in killing assays, needing 3 d for the sufficient anti-Mp effect (Figs 3 and S3).

The membrane disruption activities of AMPs seemed so weak that the activities cannot kill *M*. *pneumoniae* independently. In the colorimetric assays, the muddy precipitation occurred when effective, which indicate the sequestration of AMPs as a resistant reaction (Figs 2F, 2G, 2J, 4B, 4D, 4F, and 4H). In addition to the sequestration, there are three ways for AMP resistance: the production of exoprotease, alternation of membrane components, and efflux pumps [32]. The effect of exoprotease can be denied because Block which consists of by natural amino acids showed anti-Mp affect by killing assay (Fig 3). Alternation of membrane components can be also denied because these factors are peptidoglycan, Lipid II, or lipopolysaccharides. Efflux pumps cannot expel the molecule which attacks from outer of the cells. Before forming muddy precipitation, *M*. *pneumoniae* have to persist with the membrane damage by AMPs without protection, indicating the relatively weak activity of membrane disruption for anti-Mp effects.

The muddy precipitation was formed at the sub-anti-Mp concentrations of AMPs, which suggested molecular recognitions (Figs 2F, 2G, 2J, 4B, 4D, 4F, and 4H). Maria-Neto et al reported proteome analysis of the Magainin 1 resistant *E*. *coli*, which suggested any intracellular targets of Magainin 1 related to energy metabolism as well as bacterial membrane. If an intercellular target exists, the membrane disruption activity helps the penetration into the cells to reach the intracellular targets to enhance the anti-Mp effect. Given the reported effect of NK2A to *M*. *bovis*, *H*. *somni*, *P*. *multocida*, and *M*. *haemolytica*, the complete loss of the anti-Mp effect indicates the intracellular targets which were in those bacteria but not in *M*. *pneumoniae* [19]; the delay anti-Mp effects of Block and 17base-Hybrid also seemed to support the intracellular targets (Figs 3 and S3). The anti-Mp effects for 17base-Hybrid and Block, and the loss of anti-Mp effect for NK2A could be explained by the existence of intracellular targets. Anyhow, further studies are needed for the detailed mechanism of these antimicrobial peptides.

## Conclusions

To develop AMPs for combating the AMR of *M*. *pneumoniae*, we used the conventional colorimetric, biofilm, and killing assays to evaluate the anti-Mp effects of the characterised AMPs, Mag2, Mag2-17base, 17base-Aib, 17base-Ac_6_c, 17base-Hybrid, Stripe, Random, Block, and NK2A. The anti-Mp effects were evaluated by biofilm and killing assays, because precipitation inhibited the measurement of absorbance in the colorimetric assay. Among the tested AMPs, 17base-Ac_6_c, 17base-Hybrid, and Block showed anti-Mp effects, which appeared bacteriostatic, but NK2A, which has anti-M. bovis effect, showed limited effect against *M*. *pneumoniae*. PI uptake assays showed that 17base-Ac_6_c, 17base-Hybrid, Block, and NK2A have membrane disruption activities, indicating the cationic moiety at the terminal of AMPs as well as alpha-helical structure stability is important for membrane disruption activity; however, based on the PI uptake assay results and previous reports [11–13], although the higher membrane disruption activity basically relates to the anti-Mp effect, the AMP sequences would also affect, indicating the intracellular targets. In summary, the AMP features with terminal cationic moiety and stabilised alpha-helical structure could be potent novel antimicrobials for *M*. *pneumoniae*, but further analysis is needed for seeking the intracellular targets of AMPs. These findings would be helpful for the development of AMPs for *M*. *pneumoniae*, leading to the potent AMPs having a narrow spectrum, which can distinguish *Mycoplasma* spp.

## Supporting information

S1 Fig The quality information of the synthesized peptides used in this study. To assess the quality of the synthesized antimicrobial peptides Mag2 (A), Mag2-17base (B), 17base-Aib (C), 17base-Ac_6_c (D), 17base-Hybrid (E), Block (F), Stripe (G), Random (H), and NK2A (I), we performed Liquid Chromatograph-High Resolution Mass Spectrometry (LC-HRMS) analysis. The peptide name, length, sequence, calculated mass, its purity calculated from the area under curves with the objective by a LC-HRMS analysis, a result of the LC-HRMS, and a mass spectrum of each peptide were shown. Antimicrobial peptides were used for experiments after vacuum lyophilization.
(PDF)

S2 Fig Circular dichroism (CD) spectral analysis of antimicrobial peptides used in this study. To analyse the secondary structures of Mag2 (A and B), Mag2-17base, 17base-Aib, 17base-Ac_6_c, 17base-Hybrid (A), Block, Stripe, Random, and NK2A (B) in 20 mmol/L phosphate-based saline with 1% sodium dodecyl sulfate, 100 mmol/L of antimicrobial each peptide was used for CD spectral analysis.
(TIF)

S3 Fig Evaluation of the anti-*Mycoplasma* pneumoniae (anti-Mp) effects using the killing assay with 1 h treatment of sparfloxacin (SPFX) or antimicrobial peptides (AMPs). *Mycoplasma pneumoniae* FH were treated with 0.25, 0.125, and 0.0625 μg/mL SPFX, 30 μmol/L of AMPs (Mag2, Mag2-17base, 17base-Aib, 17base-Ac_6_c, 17base-Hybrid, Block, Stripe, Random, or NK2A), or saline (negative control) for 1 h, and inoculated onto PPLO agar in triplicate. The CFU values were determined as the number of surviving cells after 7 days incubation. Error bars indicate ± standard deviations. No statistical significance (*p* < 0.005) was found in these assays.
(TIF)
