# Supplementary material for: The effects of magainin 2-derived and rationally designed antimicrobial peptides on Mycoplasma pneumoniae
Source: PLoS One. 2022 Jan 24;17(1):e0261893. doi: 10.1371/journal.pone.0261893 (PMC8786148; doi:10.1371/journal.pone.0261893)

(A) Magainin2 (Mag2), 23 amino acids

H-GIGKFLHSAKKFGKAFVGEIMNS-NH_2

HRMS (ESI^+) calculated for $C_{114}H_{181}N_{31}O_{28}S[M+4H]^{4+}$: 616.4878; found: 617.2649.

Purity: 98.0%

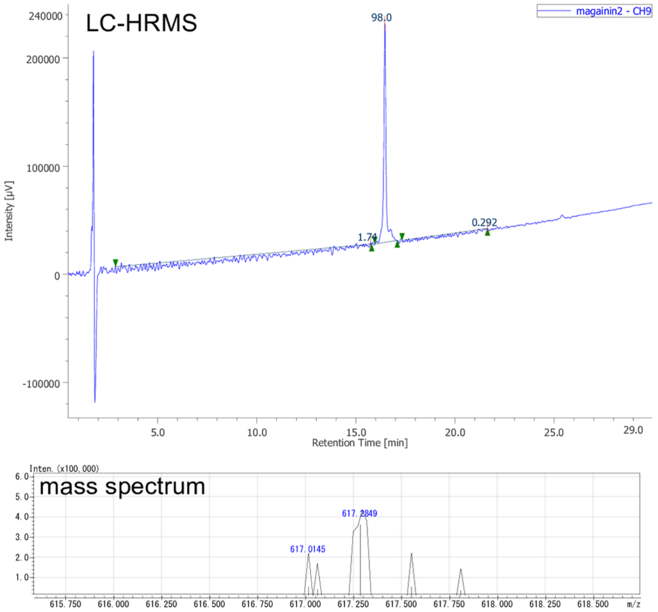

(B) Mag2-17base, 17 amino acids

H-GIGKFLHSAKKFGKAFV-NH_2

HRMS (ESI^+) calculated for $C_{89}H_{140}N_{24}O_{18}[M+4H]^{4+}$:459.2373; found: 459.4874.

Purity: 99.8%

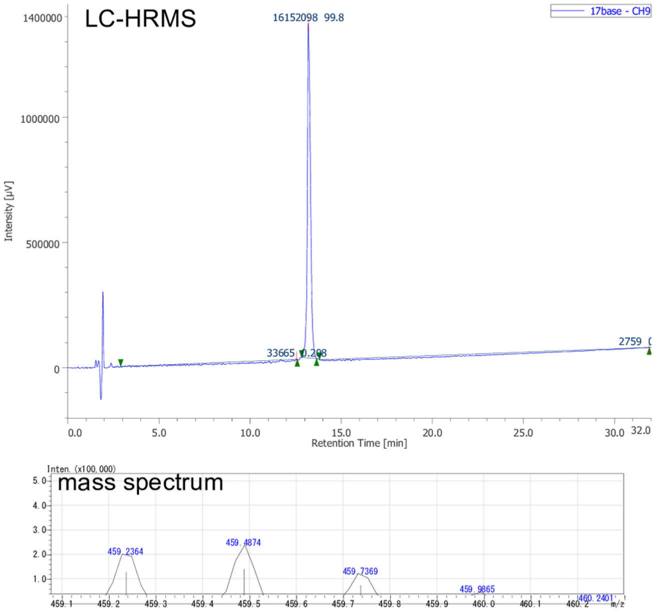

(C) 17base-Aib, 17 amino acids

H-GIGKFLHS(Aib)KKFGK(Aib)FV-NH_2

HRMS (ESI^+) calculated for $C_{91}H_{144}N_{24}O_{18}[M+4H]^{4+}$:466.2853; found: 466.5357.

Purity>99.9%

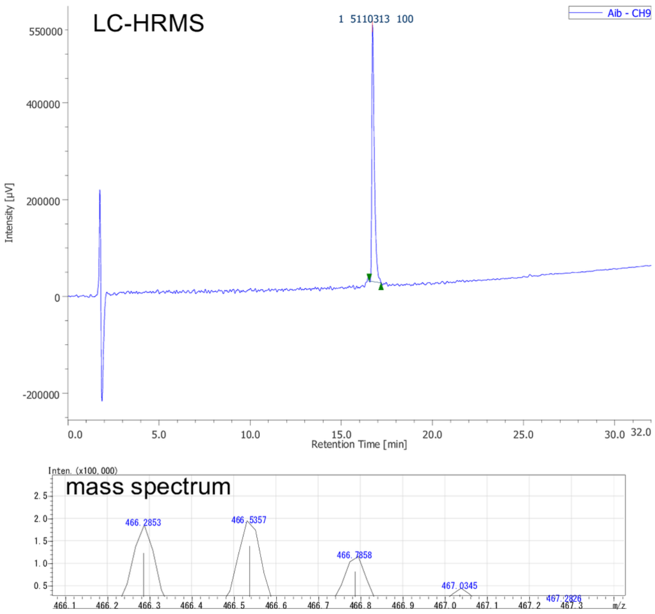

(D) 17base-Ac_6c, 17 amino acids

H-GIGKFLHS(Ac_6c)KKFGK(Ac_6c)FV-NH_2

HRMS (ESI^+) calcluated for $C_{97}H_{152}N_{24}O_{18}[M+4H]^{4+}$:486.3005; found: 486.5142.

Purity: 96.9%

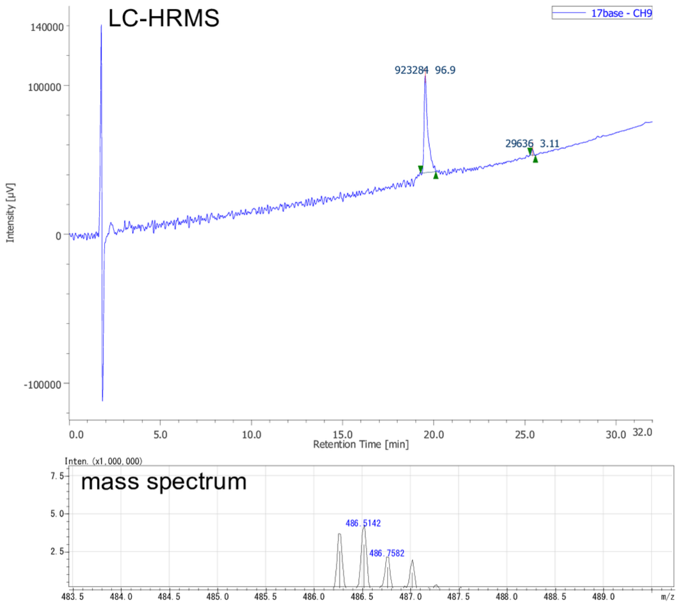

(E) 17base-Hybrid, 17 amino acids

H-GIKKFLKS(Ac$_6$c)KKFVK(Ac$_6$c)FK-NH$_2$

HRMS (ESI$^+$) calculated for $C_{97}H_{152}N_{24}O_{18}$[M+4H]$^{4+}$:486.3005; found: 486.5142.

Purity: 96.9%

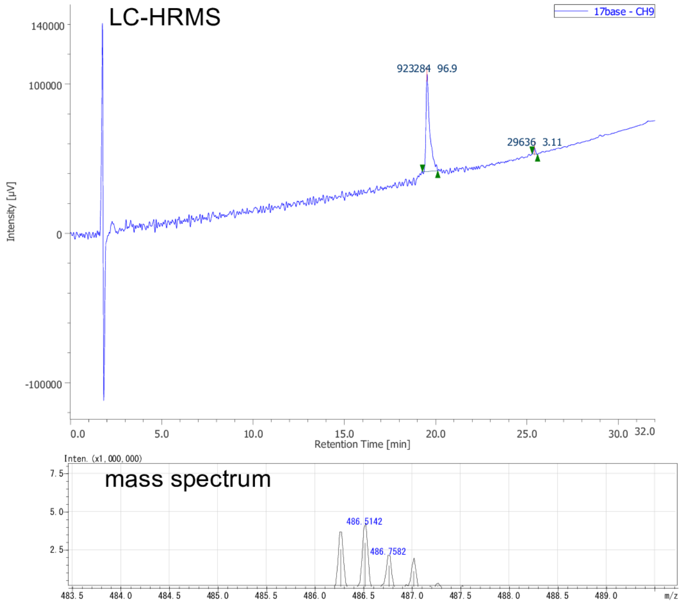

(F) Block, 21 amino acids

H-KKKKKKKKKGGGLLALLALLA-NH$_2$

HRMS (ESI$^+$) calculated for $C_{105}H_{201}N_{31}O_{21}$ [M+5H]$^{5+}$: 446.782; found: 447.4868.

Purity: 98.0%

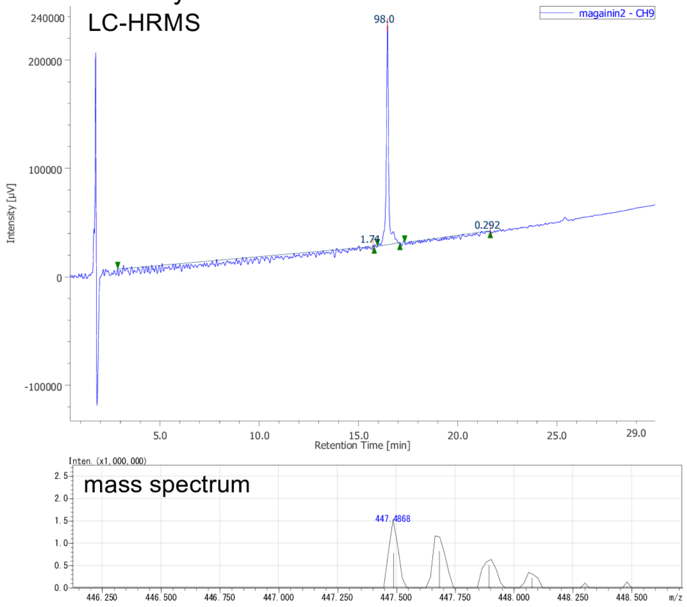

(G) Stripe, 21 amino acids

H-KLLKKAGKLLKKAGKLLKKAG-NH$_2$

HRMS (ESI$^+$) calculated for $C_{105}H_{201}N_{31}O_{21}$ [M+5H]$^{5+}$: 446.782; found: 447.6799.

Purity>99.9%

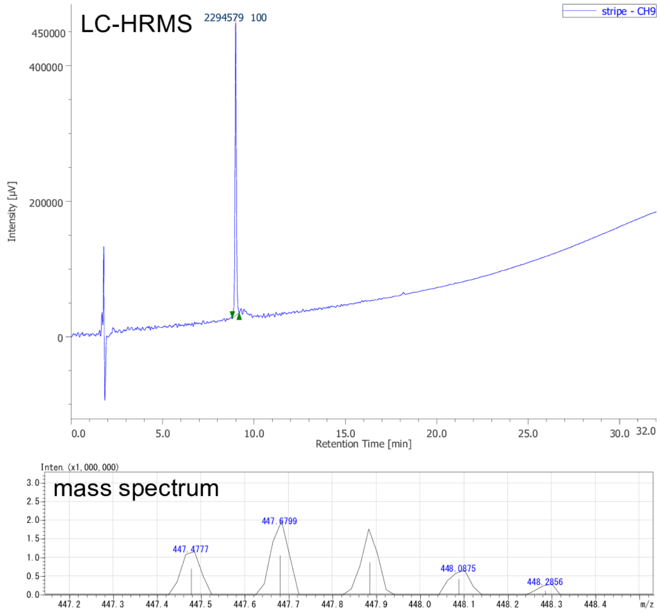

(H) Random, 21 amino acids

H-KKKLAKLKLGAKLKLKGKLGA-NH$_2$

HRMS (ESI$^+$) calculated for $C_{105}H_{201}N_{31}O_{21}$ [M+1H]$^{5+}$: 446.782; found: 447.6768.

Purity>99.9%

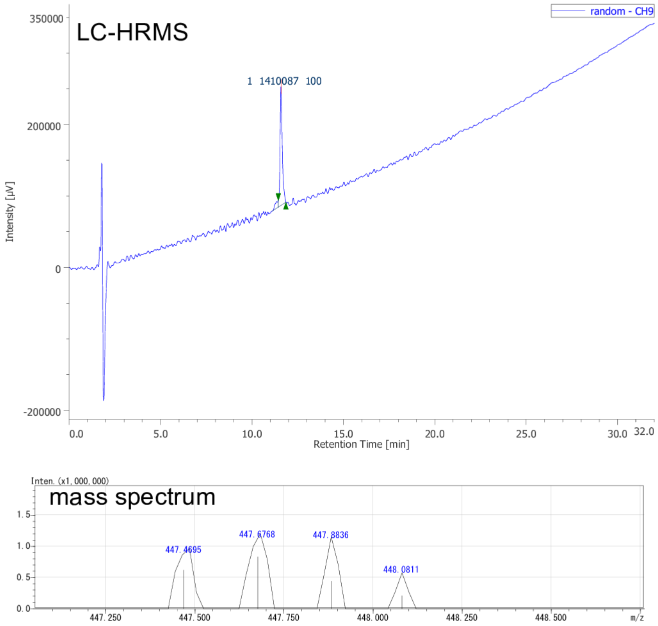

(I) NK2A, 30 amino acids

H-TVIEVASKMCSKMRLLKGLCKSITKRFLRR-NH_2

HRMS (ESI^+) calculated for $C_{152}H_{276}N_{48}O_{37}S_4$ $[M+1H]^{7+}$:500.1517; found: 500.3893

Purity>96.5%

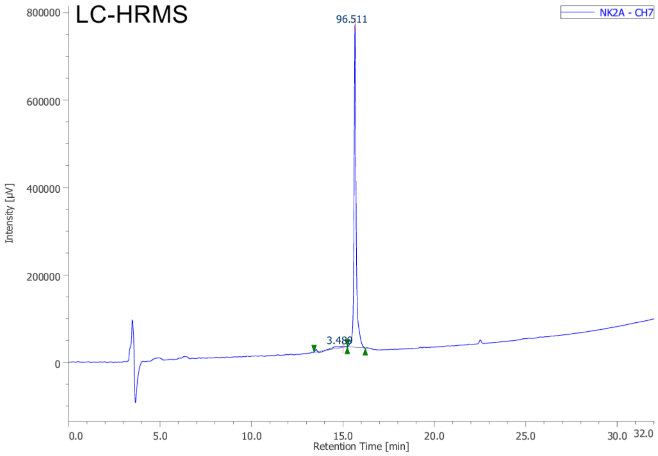

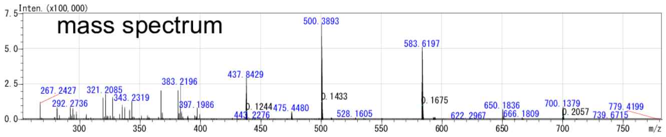

Supplement: S1 Fig — To assess the quality of the synthesized antimicrobial peptides Mag2 (A), Mag2-17base (B), 17base-Aib (C), 17base-Ac6c (D), 17base-Hybrid (E), Block (F), Stripe (G), Random (H), and NK2A (I), we performed Liquid Chromatograph-High Resolution Mass Spectrometry (LC-HRMS) analysis. The peptide name, length, sequence, calculated mass, its purity calculated from the area under curves with the objective by a LC-HRMS analysis, a result of the LC-HRMS, and a mass spectrum of each peptide were shown. Antimicrobial peptides were used for experiments after vacuum lyophilization. (PDF) [file pone.0261893.s001.pdf]
