# Supplementary figures and images for: The effects of magainin 2-derived and rationally designed antimicrobial peptides on Mycoplasma pneumoniae
Source: PLoS One. 2022 Jan 24;17(1):e0261893. doi: 10.1371/journal.pone.0261893 (PMC8786148; doi:10.1371/journal.pone.0261893)

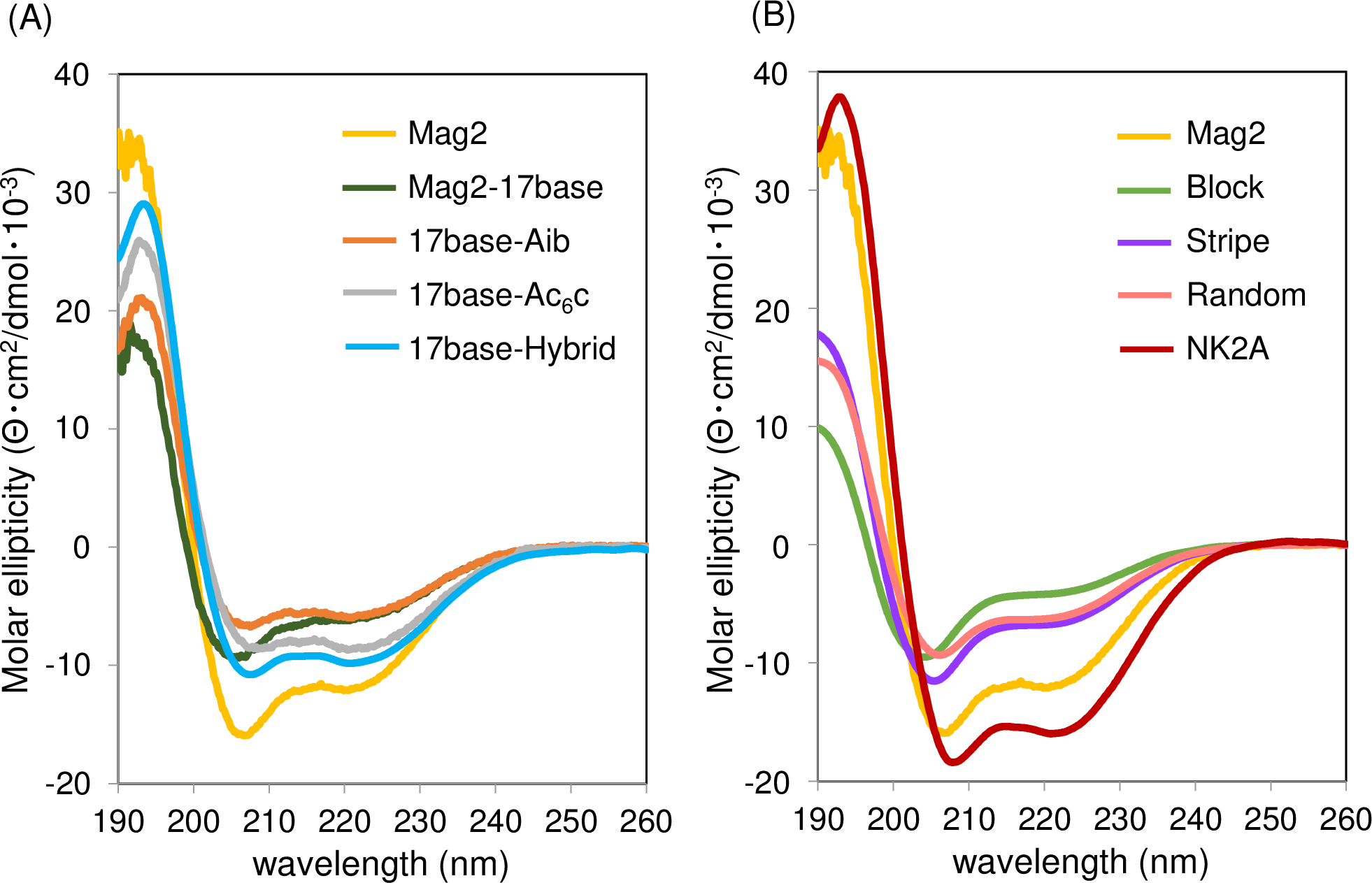

Supplement: S2 Fig — To analyse the secondary structures of Mag2 (A and B), Mag2-17base, 17base-Aib, 17base-Ac6c, 17base-Hybrid (A), Block, Stripe, Random, and NK2A (B) in 20 mmol/L phosphate-based saline with 1% sodium dodecyl sulfate, 100 mmol/L of antimicrobial each peptide was used for CD spectral analysis. (TIF) [file pone.0261893.s002.tif]

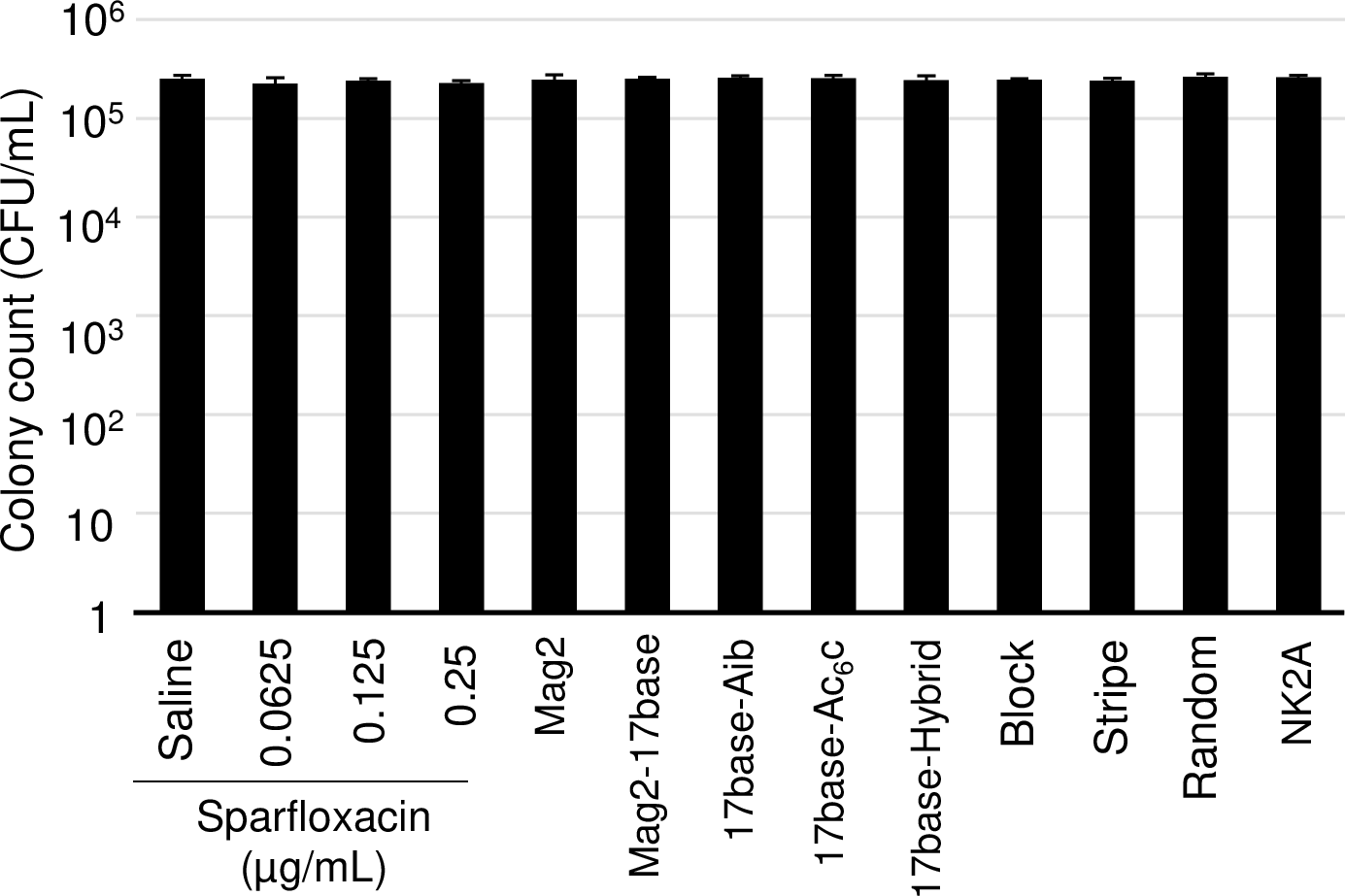

Supplement: S3 Fig — Mycoplasma pneumoniae FH were treated with 0.25, 0.125, and 0.0625 μg/mL SPFX, 30 μmol/L of AMPs (Mag2, Mag2-17base, 17base-Aib, 17base-Ac6c, 17base-Hybrid, Block, Stripe, Random, or NK2A), or saline (negative control) for 1 h, and inoculated onto PPLO agar in triplicate. The CFU values were determined as the number of surviving cells after 7 days incubation. Error bars indicate ± standard deviations. No statistical significance (p < 0.005) was found in these assays. (TIF) [file pone.0261893.s003.tif]
